# Astrocyte regulation of extracellular space parameters across the sleep-wake cycle

**DOI:** 10.3389/fncel.2024.1401698

**Published:** 2024-06-26

**Authors:** Sandhya Sriram, Kaira Carstens, Wayne Dewing, Todd A. Fiacco

**Affiliations:** ^1^Interdepartmental Graduate Program in Neuroscience, University of California, Riverside, Riverside, CA, United States; ^2^Department of Biochemistry and Molecular Biology, University of California, Riverside, Riverside, CA, United States; ^3^Undergraduate Major in Neuroscience, University of California, Riverside, Riverside, CA, United States

**Keywords:** astrocyte volume, perivascular space, glymphatic clearance, somnogen, potassium, cerebrospinal fluid, extracellular space (ECS), consciousness

## Abstract

Multiple subfields of neuroscience research are beginning to incorporate astrocytes into current frameworks of understanding overall brain physiology, neuronal circuitry, and disease etiology that underlie sleep and sleep-related disorders. Astrocytes have emerged as a dynamic regulator of neuronal activity through control of extracellular space (ECS) volume and composition, both of which can vary dramatically during different levels of sleep and arousal. Astrocytes are also an attractive target of sleep research due to their prominent role in the glymphatic system, a method by which toxic metabolites generated during wakefulness are cleared away. In this review we assess the literature surrounding glial influences on fluctuations in ECS volume and composition across the sleep-wake cycle. We also examine mechanisms of astrocyte volume regulation in glymphatic solute clearance and their role in sleep and wake states. Overall, findings highlight the importance of astrocytes in sleep and sleep research.

## 1 Introduction

Throughout the central nervous system (CNS), extracellular fluid composition has a prominent influence on neuronal activity patterns and, ultimately, the behavioral state of the organism. A delicate balance of water, ions, neuromodulators, neurotransmitters, and immunomodulators in the extracellular space (ECS) must be established and carefully controlled – a function mostly attributed to glial cells (Simard and Nedergaard, 2004; Syková, 2004; Hladky and Barrand, 2014). Given their close spatial orientation to neuronal synapses and vasculature, and the expansive array of channels, transporters, and receptors they express, astrocytes are well positioned to control ion homeostasis and other ECS characteristics (Kim et al., 2015; Haidey et al., 2021). The composition and movement patterns of cerebrospinal fluid (CSF) change dramatically over the course of the sleep-wake cycle, reflecting the shifts in activity states of both neurons and astrocytes, as well as astrocyte morphology (Ding et al., 2016; Sherpa et al., 2016; Forsberg et al., 2022). Additionally, CSF parameters are frequently impacted in several disease states that also feature sleep disruptions, and fluid dysregulation may be the factor linking these comorbidities (Taoka and Naganawa, 2021). Despite a growing body of literature highlighting the contributions of glia in sleep onset and regulation, a number of unknowns remain regarding the mechanisms by which astrocytes contribute to these changes in CSF parameters and overall state shifts.

### 1.1 Physiology of sleep and mechanisms of sleep drive: a glial perspective

The cognitive and physiological consequences of sleep loss have led researchers to conclude that sleep subserves vital maintenance functions, including waste clearance from the brain (Mendelsohn and Larrick, 2013; Xie et al., 2013; Lewis, 2021). Sleep states are distinguished from wakefulness based on the predominant frequency of cortical neuronal activity, measured by electrocorticography (ECOG) or electroencephalography (EEG), although oscillatory activity is also exhibited by subcortical structures, like the thalamus, hypothalamus, and brainstem (Halassa et al., 2010; Saper and Fuller, 2017; Lewis, 2021). The onset of sleep is typically characterized by the transition from high frequency, relatively desynchronized activity toward synchronized, large amplitude, slow wave activity (SWA), which is the predominant oscillatory pattern that characterizes non-rapid eye movement (NREM) sleep (Vaidyanathan et al., 2021). Over the course of NREM sleep, EEG patterns gradually shift toward slower oscillations that span between defined frequency ranges, with the deepest sleep characterized by delta waves (1–3 Hz) that are thought to be generated by thalamocortical circuitry (Fuller et al., 2006; Saper et al., 2010). Increased sleep intensity is associated with increased time spent in SWA and incidence of delta waves, and disruptions to NREM sleep have significant consequences for cognitive function and overall brain health (Halassa et al., 2009; Ju et al., 2017). From the deepest point of NREM sleep, oscillations abruptly transition toward rapid eye movement (REM) sleep, characterized by higher frequency activity, known as theta oscillations (4–8 Hz).

**FIGURE 1:**
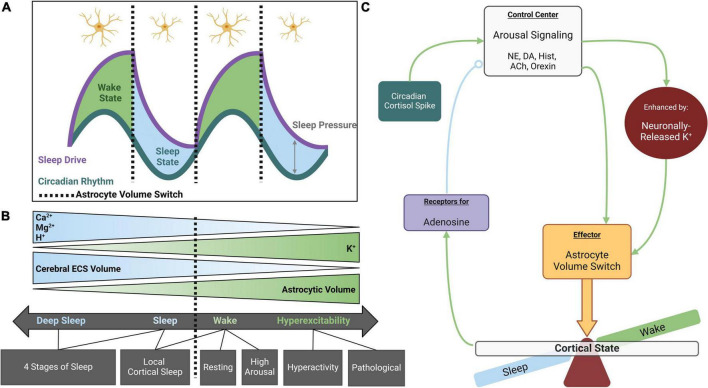
Astrocyte volume changes during sleep and wakefulness. **(A)** The sleep-wake cycle is regulated by parallel systems. Circadian rhythm fluctuates throughout the day, reaching maximal and minimal values at the midpoint of wake and sleep states, respectively. Sleep drive slowly rises to its maximum throughout the wake state and diminishes to its minimum during the sleep state. Sleep pressure is defined as the distance between circadian rhythm and sleep drive when aligned for time of day. Sleep pressure peaks just prior to entering the sleep state and is at its lowest upon entering wakefulness (adapted from Borbély, 1982). Astrocytes appear to respond to maximal and minimal sleep pressure values, rapidly adjusting their volume between sleep vs. wake states. **(B)** Astrocyte volume is inversely coupled to cerebral ECS volume such that increased astrocyte volume during the wake state is associated with decreased ECS volume. These volume changes are osmotically driven and tightly coupled to extracellular K^+^ concentrations. Extracellular Ca^2+^, Mg^2+^, and H^+^ also fluctuate across the cycle, displaying higher abundance during sleep. Cortical states exist on a spectrum that coincides with these ionic concentrations, but distinct switches occur from wake to sleep and may also occur between different stages of sleep and wakefulness. **(C)** Working model for role of astrocytes in state switches: in the maintenance of sleep-wake homeostasis, cortical arousal signaling acts as a control center for cortical states. While neuromodulators like NE and DA directly stimulate astrocytes leading to process thickening (Sherpa et al., 2016), their effects are greatly enhanced by inducing neuronally released K^+^. When astrocytes pump in K^+^ and osmotically driven water, their volume rapidly expands and compaction of the ECS acts as an effector for arousal signaling to globally induce wakefulness. The awake state leads to adenosine accumulation, a somnogen enhancing sleep drive and eventually decreasing the efficacy of arousal signaling to produce a switch to the sleep state (open circle indicates negative feedback). Created with BioRender.com.

Early efforts to characterize state transitions began with basic observations that periods of prolonged wakefulness are followed by periods of heightened fatigue and reduced latency to sleep (also known as sleep rebound). However, the theory that sleep debt results in a consistently increasing drive to sleep was contradicted by reports of daily fluctuations in fatigue across a 72-h period of sleep deprivation (Borbély, 1982). This resulted in the development of a “two-process model” of sleep, which identified two parallel but distinct cycles: a homeostatic “Process S” or “Factor S” that explains the drive to sleep as a function of the amount of time spent awake, and a state-independent “Process C” that is controlled by circadian oscillators that modulate physiological conditions (Borbély, 1982; Figure 1A).

Subsequent experiments utilizing behavioral assays and extracellular fluid analyses refined the “Factor S” concept into a somnogenic model of sleep drive, which specifies a variety of sleep-inducing components, or somnogens, that build up over the course of wakefulness and are reduced during sleep (Borbély, 1982; Krueger et al., 1990). Somnogens commonly include neurotransmitters, hormones, and various pro-inflammatory cytokines (Halassa et al., 2009; Schmitt et al., 2012; Bjorness et al., 2016), although it is important to note that certain ions may also have sleep-promoting effects (Ding et al., 2016). Somnogens accumulate during waking neuronal activity, and also remain elevated throughout the awake period. The slow and persistent time course suggests that a majority of somnogens are likely not released by synaptic activity, indicating a possible role for glia in generating this buildup (Haydon, 2017). CSF flow rate is drastically increased during sleep, suggesting that, in addition to serving as a source for somnogens, astrocytes may also serve a critical purpose in re-establishing equilibrium with respect to ECS volume, accumulated ions, neurotransmitters, and other osmolytes (Xie et al., 2013). In support of this theory, recent research has identified an astrocyte-dependent system of metabolite clearance from the brain, known as the glymphatic system (Iliff et al., 2012).

“Process C,” on the other hand, utilizes a circadian timeframe that orchestrates cellular processes such as gene expression and metabolism based on peripheral cues, including external lighting, body temperature, and energy availability (Borbély, 1982; Prolo et al., 2005; Pan and Kastin, 2017). All cells within the body show circadian oscillations of some form in their cellular processes, creating the larger cyclic framework necessary to maintain circadian rhythm, and astrocytes are no exception (Prolo et al., 2005; Lananna et al., 2018). Astrocytes have been shown to play an important role in mediating rhythmicity of neurons in several structures implicated in sleep-wake transitions, including the thalamus, suprachiasmatic nucleus (SCN), the ventrolateral preoptic area (VLPO) of the hypothalamus, and multiple regions of the cerebral cortex (Lavialle and Servière, 1993; Crunelli et al., 2002; Bellesi et al., 2015; Kim et al., 2020). Specifically in the SCN, astrocyte morphology and transcription profiles show circadian rhythmicity (Lavialle and Servière, 1993; Lananna et al., 2018), and are thought to aid in the entrainment of neuronal circadian oscillators through glutamatergic and immunomodulatory activity (Leone et al., 2006; Duhart et al., 2013; Brancaccio et al., 2017).

It is important to note that the states of sleep and wake themselves are not binary/homogeneous categories – there are different levels of alertness and arousal that may occur during wakefulness, and sleep itself is a dynamic state where the frequency of neuronal oscillations fluctuates through the various phases of sleep. Thus, additional factors must also be considered with respect to the more transient changes within states that occur over the span of minutes, rather than neuromodulator accumulation or circadian shifts that span the entire day (Saper and Fuller, 2017; Lombardi et al., 2020). Astrocytes have been previously shown to contribute to the spread of high-frequency, synchronous excitatory activity in the context of seizures (Clasadonte and Haydon, 2012; Kékesi et al., 2015), but their role in physiological localized state changes is only beginning to be studied. A recent report showed that inhibition of astrocyte signaling through block of gap junctions drastically reduced generation of SWA (Szabó et al., 2017). Astrocytes are important regulators of extracellular glutamate concentration through glutamate uptake and release, although the exact mechanisms of release are under debate (Angulo et al., 2004; Sun et al., 2014; Fiacco and McCarthy, 2018). Disruptions to glutamatergic activity in some wake-promoting neuronal populations, including the supramammillary bodies and select regions of the brainstem, have detrimental effects on wakefulness and arousal (Saper and Fuller, 2017). Interestingly, activation of these neurons has also been observed during REM sleep (Saper and Fuller, 2017). Thus, rapid reorganization of active neuronal populations may be considered a third process, in addition to large-scale circadian and homeostatic shifts driving transitions between and within states.

## 2 Choroid plexus, CSF production, and barrier functions

There have been many proposed models to explain epithelial-derived fluid and solute transport that have led to the current choroid plexus epithelial (CPE) model of CSF production and ion movement. One such proposal began with Reid (1902) on epithelial-driven fluid flow in the intestines. Studies in the 1960s saw the introduction of the Three-Compartment Model (Curran and Macintosh, 1962), where a membrane highly permeable to water but only semi-permeable to solutes separates luminal space, connected to an intermediary cellular compartment that also borders a nonselective and highly permeable interstitial membrane. As solutes collect from the interstitial side into the intermediate compartment, an osmotic gradient pulls water from the luminal side into the middle compartment. Increased pressure of the middle compartment then drives water into the interstitial layer. This model did not include a cellular description of these “compartments” but has contributed to subsequent descriptions of fluid and solute transport across epithelia. The Glymphatic Hypothesis was then introduced by Iliff et al. (2012), where an emphasis on fluid circulation was proposed as vital for the removal of hydrophilic waste metabolites including amyloid beta (aβ) and other interstitial solutes.

The blood-cerebrospinal fluid barrier (BCSFB) is functionally and morphologically distinct from all other blood–brain barrier (BBB) regions in the brain and exists within the lateral, third, and fourth ventricles containing choroid plexus (CP), the anatomical regions of CSF production (Johanson et al., 2011; Hladky and Barrand, 2014). Ion-rich CSF is secreted through cuboidal epithelial cells of the CP, each bound by tight junctions of claudin and occludin proteins, surrounding stromal cores of fenestrated capillaries (Oresković and Klarica, 2010). CP epithelium anatomy consists of an apical-basolateral cellular polarity, where an apical surface dense with secretory microvilli faces outward into the luminal space of the CSF filled ventricles, and at the other pole, the basolateral surface is directly congruent with the blood containing capillaries (Johanson et al., 2011). The ependyma contains a variety of channels and transporters that drive the passage of ions, such as K^+^, Na^+^, HCO_3_^+^, and Cl^–^ from the blood into the ventricles (Abbott et al., 2006; Oresković and Klarica, 2010). Transmembrane sodium-potassium ATPase (NKA) are located in higher concentrations on the apical surface (Spector et al., 2015). Aquaporin, namely AQP1 water channels are also present on both the apical and basolateral surfaces (Liddelow, 2015). Within the cytoplasm, carbonic anhydrase converts water and carbon dioxide into hydrogen ions and bicarbonate, which is exported into the lumen, attracting Na^+^ ions that are also extruded via NKA, providing passive transport of water into the ventricles (Oresković and Klarica, 2010). This concentration of water and ions is hypertonic to the CP cytoplasm, which further contributes to the osmotic gradient of water from the blood into the ventricles as ultrafiltrate CSF. From the ventricles, CSF flows through the subarachnoid space over the cortex, where it accesses the parenchymal space along periarterial channels (Rennels et al., 1985; Benveniste et al., 2017). This space is bordered on one side by vasculature, and on the other side by astrocyte endfeet, and it is in these spaces that arterioles make their contribution to ECS composition through the formation of interstitial fluid (ISF) (Benveniste et al., 2017).

Beyond CSF production and secretion, the CP has recently been identified as an important circadian clock (Quintela et al., 2018). Robust expression of period circadian clock 2 gene (*Per2*) (Kim et al., 2018) has been identified in CP epithelial cells, where independent oscillations mediated through gap junction (GJ) coupling enter the CSF-containing ventricles, directly influencing the SCN and circumventricular organs. Targeted deletion of CLOCK:BMAL1, a transcription factor promoting *Per2* prevented its expression within CP epithelia (Myung et al., 2018). The CP’s role as a circadian oscillator suggests that the SCN is not a top-down mediator of downstream circadian rhythms, and that peripheral non-neural CP *Per2* oscillations may contribute a larger role in coordinating circadian cycles. Changes in ventricular architecture and CSF movement during sleep are congruent with CP *Per2* production as a peripheral mediator of circadian patterns.

## 3 Astrocytes as drivers of water and solute transport during sleep

Astrocytes exert considerable influence over fluid distribution through the brain, carrying water, ions, and neurotransmitters that support neuronal function (Kimelberg et al., 1990; Kofuji and Newman, 2004). This fluid can be broken down into two distinct but related types – CSF, the fluid that surrounds the entire brain and flows through the ventricular system, and ISF, the fluid that passes through the brain parenchyma, carrying ions, and other components through the neuropil and clearing away metabolites as they accumulate from neuronal activity (Louveau et al., 2017; Hablitz and Nedergaard, 2021; Thomas, 2022). In addition to the ionic and neurotransmitter components of CSF and ISF, the ECS is also a site of accumulation for cellular waste products that may be toxic in high concentrations, including lactate and protein aggregates (Xie et al., 2013; Kress et al., 2014). These substances are known to build up over the course of wakefulness, and their clearance from the ECS relies on astrocyte-mediated exchange of CSF and ISF through the periarterial and perivenous spaces that, together, make up the glymphatic system (Iliff et al., 2012). The efficiency of this clearance is improved during sleep, both due to slower buildup of solutes, and to an increase in ECS volume fraction (Xie et al., 2013). Thus, in addition to modulating ion fluctuations that subserve sleep drive and sleep architecture, astrocytes are also well-equipped to facilitate the restorative properties of sleep.

### 3.1 Astrocyte volume and morphology changes during state transitions

Consensus exists within the field regarding the role of astrocytes as effectors of arousal signaling (Pacholko et al., 2020). Astrocyte volume change coincides with the maximum and minimum of sleep drive, where sleep pressure is also at its maximum and minimum, and state switches occur (Figure 1A). Regulation of CSF components, specifically K^+^, controls cerebral ECS volume and neuronal excitability (Figure 1B; Amzica et al., 2002; Rasmussen et al., 2019; Walch et al., 2022). ECS volume is inversely proportional to neuronal excitability such that expansion of the ECS, which ultimately decreases cortical tortuosity allowing for “washing” of ISF, is associated with the sleep state (Xie et al., 2013; Tuura et al., 2021; Thomas, 2022). This space then shrinks upon entering wakefulness (Haj-Yasein et al., 2012; Ding et al., 2016; Sherpa et al., 2016). Astrocyte morphology and *ex vivo* experimentation favor astrocytes as a key contributor to ECS volume dynamics (Lauderdale et al., 2015; Reed and Blazer-Yost, 2022; Walch et al., 2022). While cellular volume is not dichotomous, rather existing on a continuum, distinct stages of this spectrum may also occur within cortical states. State changes are associated with an alteration of CSF and ISF composition (Rasmussen et al., 2019; Forsberg et al., 2022), which are subsequently accompanied by alterations in astrocyte and ECS volume (Simard and Nedergaard, 2004; Neprasova et al., 2007).

**FIGURE 2:**
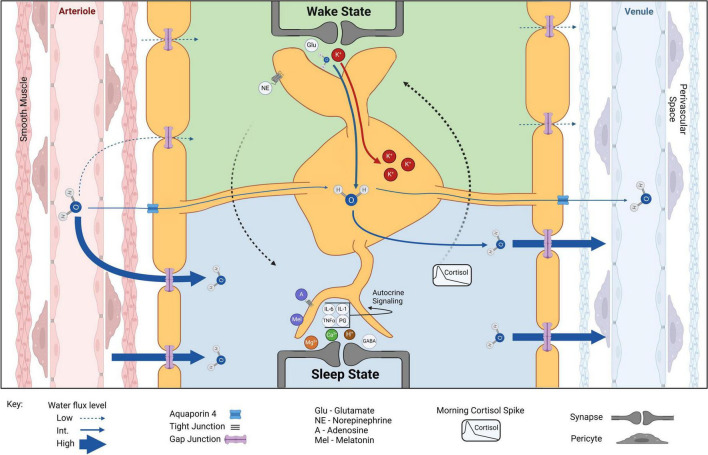
Astrocytic volume and CSF component shifts from wake to sleep. Water exits arterioles through tight junctions, enters the perivascular space before passing between smooth muscle cells, and finally enters the brain through or between the syncytium of astrocyte endfeet, facilitated by AQP4. Wake state: during wakefulness, arousal signaling (e.g., NE) dominates and extracellular glutamate (Glu) and K^+^ concentrations increase. Accumulation of K^+^ and water into astrocytes increases astrocyte volume and decreases overall ECS. Astrocytes help monitor and maintain vascular tone, allowing for localized increases in cerebral blood flow coupled specifically to local changes in cortical activity. Wake-sleep switch (dotted arrows): long-term arousal signaling and circadian rhythm increase somnogen concentrations (e.g., A, adenosine; Mel, melatonin) increasing sleep drive and eventually switching the system to a sleep state. Sleep state: extracellular K^+^ decreases in sleep result in reduced astrocyte volume and increased ECS. Water flux through brain parenchyma is greatly favored in the sleep state. Extracellular Mg^2+^, Ca^2+^, and H^+^ are increased but do not lead to osmotic increase in astrocyte volume. Increased extracellular GABA and Mg^2+^ concentrations inhibit cortical activity while increased extracellular Ca^2+^ indicates decreased Ca^2+^ activity within cells. A slight increase in H^+^ may result from ECS volume increase and effective reduction of carbonic anhydrase levels. Sleep-wake switch (dotted arrows): circadian-driven early morning cortisol spike reengages arousal signaling to induce ECS and astrocyte volume conducive to the wake state. Created with BioRender.com.

Astrocyte *morphology* during wakefulness is also distinct from the sleep state regarding increased process number, expansion, and proximity to the synaptic clefts (Figure 2; Bellesi et al., 2015; Sherpa et al., 2016). The drive for this mechanism is attributed to the need for astrocytic removal of K^+^ during wakeful neuronal activity. The importance of astrocytic regulation of extracellular K^+^ is well established in the literature (reviewed in Kofuji and Newman, 2004). While local increases occur directly after neuronal activity (Barron and Kim, 2019), arousal signals such as norepinephrine (NE) globally elevate extracellular K^+^ *in vivo* through an independent pathway (Ding et al., 2016; Rasmussen et al., 2019). Elevated extracellular K^+^ produces intracellular K^+^ accumulation generating osmotically driven astrocyte volume increase (Walz, 2000; MacVicar et al., 2002; Walch et al., 2022). Evidence suggests this K^+^ entrance into the cell is mainly via the NKA (Wang et al., 2012; Larsen et al., 2014; Walch et al., 2020), which is enhanced by adrenergic stimulation (Hertz et al., 2015), leading to intracellular K^+^ accumulation and increased astrocytic volume during wakefulness. This astrocytic activity decreases ECS volume, increases ambient neurotransmitter concentrations, and enhances point-to-point synaptic transmission involving lower affinity receptors during wakefulness.

Conversely, extracellular K^+^ is significantly lower during sleep (Ding et al., 2016; Rasmussen et al., 2019). This reduction is not simply due to the dilution of ISF; rather, a specific reduction in K^+^ has been observed at sleep onset in healthy humans (Forsberg et al., 2022). Astrocytes respond to the reduction in extracellular K^+^ with both a reduction of intracellular K^+^ accumulation and a reduction in osmotically driven water influx (Pasantes-Morales and Schousboe, 1989; Walz, 2000; MacVicar et al., 2002; Walch et al., 2022), allowing for widening of the ECS and facilitated ISF movement through brain parenchyma throughout sleep (Xie et al., 2013; Tuura et al., 2021; Thomas, 2022). One foundational question remains: how is extracellular K^+^ reduced while we sleep? While a small proportion may be cleared systemically through the bloodstream (Bradbury and Stulcová, 1970), neurons could also be potential candidates for K^+^ storage during sleep, as they are significantly less prone to osmotic fluctuations to the same extent as astrocytes (Hellas and Andrew, 2021). The mechanisms of nightly ion fluctuations, as well as how these fluctuations affect astrocyte volume, require further investigation.

### 3.2 Arousal signaling, Ca^2+^, and adenosine

Cortical arousal signaling, integrating NE/dopamine (DA)/histamine/acetylcholine/orexin pathways, paradoxically modulates wake and sleep states (Figure 1C). Immediate effects of arousal signaling are as expected: heightened release of neuromodulatory neurotransmitters increases cortical excitability in the wake state. In addition to direct neuronal stimulation, adrenergic receptor activation on astrocytes directly modulates astrocyte volume via process expansion which concurrently reduces ECS volume (Sherpa et al., 2016) – anatomy reflective of the wake state. However, downstream of these immediate effects lies a negative feedback system wherein increased arousal signaling raises the probability of eventual cortical state switching to sleep (Poskanzer and Yuste, 2011). Therefore, it could be suggested that classification of arousal signaling as strictly wake-promoting is only part of the story, and a more fitting classification may be as a regulator of sleep homeostasis.

A decrease in extracellular Ca^2+^ is associated with the wake state (Ding et al., 2016; Ingiosi et al., 2020), which is an arrangement conducive to greater Ca^2+^ signaling within astrocytes and neurons which has been observed during wakefulness (Clapham, 2007; Ingiosi et al., 2020). Notably, this astrocytic Ca^2+^ activity is attenuated by anesthesia (Thrane et al., 2012), indicating a connection with cortical states. Paradoxically, studies have linked the rise in astrocytic Ca^2+^ activity during the wake state with downstream mechanisms eventually promoting sleep. In fact, it has been proposed that levels of astrocyte Ca^2+^ signaling during the wake state are proportional to, or code for, sleep need (Ingiosi et al., 2020). Some have found this astrocytic signaling to modulate the REM stage of sleep, specifically (Foley et al., 2017).

While most studies focus on NE activation of adrenergic receptors increasing astrocytic Ca^2+^ signaling (Paukert et al., 2014; O’Donnell et al., 2015), recent work also implicates DA as an agonist for at least one adrenergic receptor class (Pittolo et al., 2022), and serotonin (5HT) directly increases astrocyte Ca^2+^ signaling (Schipke et al., 2011) by working on 5-HT2B or 5-HT2C receptor subtypes (Natsubori et al., 2023). It is important to note that astrocyte output behavior in response to increased Ca^2+^ activity is likely based on location. In some astrocyte populations, transient Ca^2+^-stimulated glutamate release increases prior to local neurons switching to the sleep state (Poskanzer and Yuste, 2016). Astrocytic redistribution of glutamate from perisynaptic to simple parenchymal spaces surrounding the astrocyte appears to effectively induce sleep locally in the population of neurons it interacts with. It has been hypothesized that the cytosolic syncytium of astrocytes may be crucial for spreading this sleep signal to other areas (Poskanzer and Yuste, 2016).

Global NE from locus coeruleus (LC) neurons not only contributes to changes in astrocytes conducive to the wake state of neural circuitry, but also induces nonselective cerebral vasoconstriction despite the lack of direct communication with vasculature (Cohen et al., 1997; Bekar et al., 2012). Many studies have implicated astrocytes as the mediator of NE-induced cerebral vasoconstriction (Cohen et al., 1997; Mulligan and MacVicar, 2004; MacVicar and Newman, 2015). In this way, astrocytes mediate the excitatory state of neurons while simultaneously ensuring active circuits receive sufficient oxygen and nutrients when only their local vasculature dilates. In addition, pulsatility of cerebral blood flow in SWA during sleep is mediated by astrocyte endfeet regulation of vascular tone (Fultz et al., 2019; Marina et al., 2020). Therefore, astrocytes not only regulate the distribution of CSF components within parenchyma, but also modulate entrance of new osmolytes from systemic circulation.

Alternatively, some have found that Ca^2+^-mediated astrocytic release of ATP, which is rapidly metabolized extracellularly into adenosine, precedes synaptic depression (Pittolo et al., 2022). Because arousal signaling increases astrocytic catalysis like glycogen breakdown (Coggan et al., 2018), a metabolic accumulation of adenosine (a suggested somnogen) could also result in an eventual switch to the sleep state when the threshold for sleep drive is surpassed, bringing sleep pressure to its maximum (Figures 1A, C). By monitoring adenosine accumulation through an adrenergic homeostatic mechanism, it appears that astrocytes may monitor the energy availability of brain tissues and sustainability of a wakeful state (Agostinho et al., 2020; Garcia-Gil et al., 2021). According to the “Process S” model proposed by Borbély (1982), the drive for sleep would increase as the probability of efficient cortical processing decreases. Somnogen levels and sleep drive then decrease over the course of sleep as ISF washes through the parenchyma.

Cortisol peaks early in the morning (Krieger et al., 1971; Weitzman et al., 1971; Oster et al., 2017) and acts on the LC to increase global NE release (Wang et al., 2015). This is an attractive trigger for the exit of sleep (Figures 1C, 2). Upon direct NE activation, astrocyte volume increases and Ca^2+^ activity may play a role in initiating the wake state. A simultaneous feedforward mechanism may then accelerate this switch: NE-activated neurons release K^+^ into the ECS and an osmotically driven increase in astrocyte volume occurs. This could partially explain the phenomenon of sleep paralysis where subjects are awoken, immobile, and often terrified (Stefani and Högl, 2021). If night terrors in REM increase cortisol past the threshold for stimulating arousal signaling, but somnogens and other sleep-promoting factors have not yet fully cleared from brain parenchyma, a partially wakeful state could result while local cortical areas (like motor cortex) remain in the sleep state.

### 3.3 Contributions of gap junctions to movement of fluid and ions between astrocytes

Gap junctions, or electrical synapses are intercellular protein pores composed of apposing hexameric hemichannels, or connexons in chordates, directly connecting the cytoplasm of one cell to the cytoplasm of another. Unlike the 20–30 nm synaptic cleft observed with chemical synapses, the electrical synapse is an order of magnitude smaller, with respective cell membranes roughly 2–3 nm apart (Goodenough and Paul, 2009). Each connexon is composed of six individual connexin (Cx) protein subunits (Eiberger et al., 2001). Research on the murine CNS found that GJ are expressed in a number of cell types including neurons, astrocytes, and oligodendrocytes (Rash et al., 2001), as well Schwann cells in the peripheral nervous system (Cisterna et al., 2019) and most tissues throughout the body proper. With an average pore diameter of 1.4 nm, GJ permeability is constrained to the passage of small molecules roughly 1 kDa or less (Weber et al., 2004), allowing the direct exchange of water, ions, amino acids, sugars, lactate, and signaling molecules such as adenosine, ATP, cyclic AMP, cyclic GMP, and inositol trisphosphate (Veenstra et al., 1994), while excluding larger proteins or macromolecules. Formation of GJ are facilitated through three cysteine binding domains located on each of two Cx extracellular loops, 36 total domains per connexon, producing anti-parallel β-Barrel interactions along 24 total rods shared from two apposing connexon hemichannels (Unger et al., 1999), establishing the functional GJ channel. For healthy cells, this coupling conserves both electrical current and molecular exchange with virtually no loss into the ECS (Scemes et al., 2007).

Gap junctions are further organized into large densities called GJ plaques often consisting of many thousands of individual functional channels (Loewenstein, 1981). Large GJ plaque formations further contribute to inter-cell adhesion, strengthening and stabilizing syncytia (Nakagawa et al., 2010). GJ plaques are not static structures, and motility rates vary as a function of Cx composition. GJ nexus formation depends on the affinity of GJ-associated membrane proteins, either interacting within the plaque or clustering along the periphery. In the brain, Cx26, Cx30, and Cx43 are exclusively expressed in adult astrocytes (Rash et al., 2001), establishing specificity in targeting astrocyte-mediated GJ properties. Levels of Cx expression vary greatly throughout the CNS. Immunolabeling has shown high Cx30 expression in the cerebellum and thalamus, and moderate expression in the cerebral cortex, while Cx43 is abundant throughout the cerebral cortex and highly expressed in hippocampal astrocytes (Nagy et al., 2004; Gosejacob et al., 2011). Examples of GJ-associated proteins include zonula occludens-1, claudin-1, occludin, the oncogenic signaling protein Src, aquaporin-4 (AQP4), and other structural proteins including tubulin (Duffy et al., 2002). In astrocytes, Cx43 GJ plaques were shown to be considerably less motile and more stable than Cx30 GJ due to unique cysteine interactions along the cytoplasmic C-terminus domain. These cysteine interactions also attract a clustering of peripheral AQP4 channels, forming a Cx43-AQP4 peri-nexus, contributing to the anchoring of astrocyte endfeet around vasculature and the regulation of the BBB (Cibelli et al., 2021). Deletion of AQP4 in *ex vivo* hippocampal slices produced a proportional upregulation of Cx43, suggesting a compensatory mechanism for fluid regulation and K^+^ buffering, highlighting the dynamic interdependence between AQP4 and GJ in ECS homeostasis (Strohschein et al., 2011; Katoozi et al., 2017). Especially in densely organized gray matter regions, water diffusion faces greater resistance in the ECS than through astrocyte syncytia. Accordingly, the co-organization of AQP4 and GJ coupled astrocyte networks is an important means for fluid movement and metabolite clearance in the brain (Asgari et al., 2015). Human patients with chronic insomnia display loss of Cx30 and Cx43 along with decreased levels of AQP4, supporting a critical role for astrocytes in the regulation of both sleep and fluid dynamics (Yang et al., 2022).

Astrocytes use GJ to organize into large astrocyte-coupled networks throughout the CNS, maintaining ECS homeostasis through the uptake of key extracellular ions: K^+^, Na^+^, and Ca^2+^. This net uptake is followed by spatial buffering, or the long-distance redistribution of signaling ions, preventing their accumulation around neurons and triggering hyperexcitability (Orkand et al., 1966; Kofuji and Newman, 2004). Given that the maintenance of K^+^ concentration within the ECS is intimately tied to arousal signaling and ECS volume, GJ could represent a significant contribution to the redistribution of K^+^ that occurs across state transitions (Ding et al., 2016). Conversely, loss of GJ coupling as a result of inflammatory signaling has been shown to hinder K^+^ buffering, leading to tissue damage and increased incidence of seizures in a mouse model of temporal lobe epilepsy (Bedner et al., 2015). Ion uptake is maintained through the timing of cascading waves traveling throughout the network, which are ultimately expelled into the ECS and neighboring capillaries, sustaining a stable inward ionic driving force into astrocytes at active tripartite synapses where constant ECS regulation is needed (Ma et al., 2016). The astrocytic GJ network may influence sleep through the diffusion of somnogenic substances, ions, and metabolites such as lactate, across the parenchyma (Petit and Magistretti, 2016). Disruption of this network through loss of Cx43 reduces activation of orexinergic neurons responsible for wakefulness, resulting in abnormal sleep architecture, with wake periods frequently interrupted by bouts of NREM sleep (Clasadonte et al., 2017). Conversely, sleep recovery following sleep deprivation is characterized by an upregulation of Cx43, which authors speculate may reflect an attempt to counteract fatigue by increasing ATP-release mechanisms (Franco-Pérez et al., 2012). Inflammatory signaling, which is a commonly observed consequence of sleep disruption, also interferes with GJ function (Même et al., 2006; Bedner et al., 2015). Taken together, these findings suggest that GJ are well-positioned to make significant contributions to fluid composition changes and solute transport across the sleep-wake cycle, although the specific mechanisms require further investigation. Additionally, disruption to GJ-mediated signaling could prove consequential for the initiation and maintenance of behavioral states, especially wakefulness.

## 4 The glymphatic system

Significant early characterization of the glymphatic system has shown that ions and larger solutes do not simply pass through brain parenchyma through bulk diffusion; rather, they are carried throughout specific pathways around perivascular spaces, transporting a wide range of molecules through and out of the brain (Iliff et al., 2012). Commonly used methodologies to study fluid movement involve injection of inert markers, such as ^14^C-inulin, whose pathways can be radioactively traced through brain tissue. Initial observation of whole-brain coverage of ^14^C-inulin supported the hypothesis that fluid and associated solutes diffuse through the brain along a concentration gradient, known as “bulk flow” (Proescholdt et al., 1999). However, these authors acknowledged that diffusion was unlikely to be the sole force, given the rapid time course of tracer coverage, the uneven distribution of fluid to specific structures, and the penetration of the tracer into deep structures despite high levels of obstruction. This would be more characteristic of “convective flow,” or the passage of solutes along a current, implying that there must be some channel-like structure that allows fluid to carry solutes toward specific structures that would supplement transport through bulk flow (Proescholdt et al., 1999). A landmark study by Iliff et al. (2012) set out to identify these routes using high-resolution two-photon imaging of fluorescent tracers injected into the parenchyma of anesthetized mice. They found that fluid and small molecular weight dextrans enter the parenchyma from perivascular spaces that are contiguous with the subarachnoid space, bound on one side by endothelial cells and on the other by astrocyte endfeet. Subsequent experiments emphasized the importance of astrocytes by demonstrating that interfering with AQP4, the water channel highly expressed in astrocyte endfeet, compromised the penetration of tracers into the parenchyma (Iliff et al., 2012).

### 4.1 Astrocyte–vasculature interactions in glymphatic clearance

As the brain cells that interact most closely with endothelial cells, astrocytes are uniquely positioned to influence the extravasation of solutes from brain vasculature into brain parenchyma (Abbott et al., 2006; Díaz-Castro et al., 2023; Verkhratsky and Pivoriūnas, 2023). They are also capable of sensing vascular tone and perfusion pressure within cerebral arterioles (Kim et al., 2015; Marina et al., 2020). Efforts to characterize fluid distribution throughout the brain have focused on the perivascular space as the location for fluid exchange, with astrocytes pulling water and solutes out of, and driving toxic metabolites into, these compartments (Iliff et al., 2012). The pulsation of cerebral arteries is thought to be the main physical force that enables CSF from the subarachnoid space to enter the brain through deep penetrating arteries and mix with ISF in the parenchyma (Iliff et al., 2013). In testing this hypothesis, Iliff et al. (2013) showed that pharmacological reduction of this pulsation slowed influx of an inert tracer into intact brain tissue, supporting previous findings that blockage of main arteries supplying the brain reduced the rate of CSF movement (Rennels et al., 1985).

Most of the restorative functions associated with sleep, like glymphatic clearance, are thought to occur during low-frequency neuronal activity characteristic of slow-wave sleep (SWS) (Hablitz et al., 2019). Oscillatory fluctuations of vascular tone are closely tied to neuronal rhythmic activities, especially slow-frequency rhythmic activities with increased blood flow required to support increased neuronal firing (Haidey et al., 2021; Loshkarev, 2021). Simultaneous BOLD-fMRI (blood oxygenation level dependent functional magnetic resonance imaging) and CSF tracing has shown that large influxes of CSF from the fourth ventricle into the brain are temporally coupled with low-frequency neuronal activity and oscillations in blood volume in the sleeping human brain (Fultz et al., 2019). While these findings suggest that pulsatory activity of arteries could drive CSF into the parenchyma *and* into individual astrocytes, it has yet to be thoroughly established whether this pulsatory activity causes direct changes to astrocyte volume. Novel imaging techniques, such as super-resolution shadow imaging, are a promising step in understanding how CSF and ISF move, not only through the parenchyma, but also through individual astrocytes (Tønnesen et al., 2018; Arizono et al., 2021).

While much attention has been given to astrocyte–arteriole interactions for the composition of ISF, less is known regarding waste drainage through the perivenous spaces directly into cerebral venules, or through arachnoid granulations that drain into the venous sinuses and lymph (Hladky and Barrand, 2018; Hu et al., 2023). Some tracer studies support the latter, showing that the bulk of ISF outflow may be directed toward the deep cervical lymph nodes, to merge with the fluid circulating through the rest of the body (Ma et al., 2017, 2019; Liu et al., 2021). Given that fluid and solutes are expelled from periarterial spaces by the pulsating pressure of cerebral arterioles, it is possible that the colloidal osmotic pressure created by ECS solutes and parenchyma could provide the pulling force to draw ISF into perivenous spaces. The state dependency of perivenous and lymphatic drainage have only recently begun to be investigated, as are the consequences of sleep deprivation (Eide and Ringstad, 2021). The specific characteristics of astrocyte–venule interactions remain unknown, though astrocytic water channels could hold the answers to the pathways of fluid movement across the brain.

### 4.2 Role of aquaporin-4 in water and solute transport

Reduction of astrocyte volume during sleep is vital to waste clearance, providing both space and physical driving force for fluid movement in the form of various water channels and transporters (Mendelsohn and Larrick, 2013; Xie et al., 2013). Of all the putative routes of fluid entry from the vasculature into the neuropil, the water channel AQP4 has been the most thoroughly studied and widely accepted avenue (Nagelhus and Ottersen, 2013; Mader and Brimberg, 2019). The most abundantly expressed water channel in the brain is AQP4, predominantly on astrocytic endfeet that form one end of the Virchow-Robin space. The AQP4-rich endfeet of astrocytes have unique access to fluid-filled perivascular spaces, making them a robust potential mechanism for fluid intake from the arteries and subsequent drainage through the venous system (Manley et al., 2000; Reed and Blazer-Yost, 2022; Salman et al., 2022). In the context of glymphatic transport, AQP4 may be considered to serve three important functions: an entry route for water, an outlet route for water, and as a mechanism for solute transport across astrocytes (Solenov et al., 2004; Verkman et al., 2006; Jin et al., 2013; Stokum et al., 2018; Walch et al., 2020).

The advent of AQP4 knockout mice has enabled a widespread effort to characterize AQP4 expression and function, in both the healthy and edematous brain (Ma et al., 1997). For a comprehensive review of currently utilized AQP4 knockout strategies (see Mestre et al., 2018). It is important to note that these strategies have involved removal of astrocytic AQP4 constitutively throughout brain development. Inducible models of astrocytic AQP4 removal may better delineate the role of AQP4 in adult astrocytes and glymphatic clearance where possible compensatory effects could be minimized. Measurements obtained from live animals, as well as from brain slices, have shown that AQP4-deficient animals display a larger ECS volume without any change in underlying neuropil structure, suggesting that fluid entry through AQP4 does play a role in establishing initial baseline ECS volume in healthy animals without affecting astrocyte process orientation (Yao et al., 2008). Similar findings were also observed from *in vivo* recordings of AQP4 knockout mice, which show decreased water diffusion and enlarged interstitial spaces relative to AQP4 intact controls (Gomolka et al., 2023). Key glymphatic system experiments by Iliff et al. (2012) showed that CSF flow was significantly decreased in mice with global AQP4 knockout, compared to AQP4-intact controls. Pharmacological blockade of AQP4 also caused a reduction of water influx from vasculature to parenchyma, as well as a decrease in glymphatic clearance away from the brain (Giannetto et al., 2024). Conversely, use of a novel AQP4 agonist increased circulation of water throughout the ECS (Huber et al., 2018). Taken together, these findings emphasize the significance of AQP4 not only in the introduction of fluid into the parenchyma, but also in the prevention of fluid accumulation by providing a route for clearance away from the brain (Gomolka et al., 2023).

The importance of functional AQP4 activity to overall brain health can also be understood via various pathological contexts, including brain inflammation, ischemia, and sleep deprivation. Upregulation of AQP4 can be triggered by a lipopolysaccharide (LPS)-triggered immune response (Alexander et al., 2008; Cao et al., 2012; Sugimoto et al., 2015), and heightened levels of AQP4 are thought to be a key mechanism of cellular edema in models of ischemic stroke (Papadopoulos et al., 2004; Warth et al., 2007; Yang et al., 2008). Increased AQP4 expression also results in increased susceptibility to cellular edema, ECS constriction, and mortality (Yang et al., 2008). Accordingly, ablation or blockage of AQP4 has been shown to relieve some fluid accumulation within cells (Manley et al., 2000; Pirici et al., 2017; Sun et al., 2022). While the significance of AQP4 for water homeostasis in the brain has been clearly demonstrated in these studies, reliance on constitutive knockout makes it difficult to interpret whether the findings are due to AQP4 loss in the context of the experiment, or due to secondary changes to astrocyte physiology and vasculature characteristics as a consequence of AQP4 loss during brain development.

Results from a variety of experimental models and pathological conditions show that glymphatic clearance is significantly reduced when AQP4 activity or expression is compromised (Teng et al., 2018; Giannetto et al., 2024). However, the specific mechanisms behind how AQP4 fits into the cycle of fluid dysregulation and sleep disruptions observed across disease states is still unknown. Ablation of AQP4 has been reported to have both neuroprotective effects (Manley et al., 2000; Chmelova et al., 2019) and detrimental consequences (Binder et al., 2006). Taking into account contributions of AQP4 to solute concentration *and* transport in the ECS, we propose the following hypothetical framework for AQP4’s role in sleep-mediated glymphatic transport. In normal healthy conditions with physiological expression of AQP4, CSF passes into the parenchyma, merges with ISF, and is then driven through the parenchyma by pulsatile activity of intact vasculature (Iliff et al., 2012, 2013). Water, ions and solutes would move through the variety of channels expressed on astrocyte endfeet, including *but not limited to* AQP4 (Walch et al., 2020). While this is likely a continuous process that occurs to some degree across activity states, the widening of the ECS during sleep onset allows ease of movement of ISF and solute transport through the parenchyma. The ability of AQP4 to then act as an outlet for water allows for water and solute discharge from perivenous endfeet into the perivenous space to be recycled back into the subarachnoid space. Again, some of these solutes may be passed directly into the venules by channels and transporters within endothelial cells, while some may require astrocytic endfeet to facilitate their passage. Under these conditions, the upregulation of AQP4, a well-characterized phenomenon in edematous astrocytes (Vizuete et al., 1999; Warth et al., 2007; Zhang et al., 2015; Liu et al., 2021), could be considered a compensatory mechanism for expelling accumulated water from cells.

While it is important to understand factors that regulate expression of AQP4, an equally influential component of its role in the glymphatic system lies in its localization, or polarization, to perivascular astrocyte endfeet (Nielsen et al., 1997; Nagelhus et al., 1999). Endfoot AQP4 exists in multiple isoforms, which are further organized into aggregations known as orthogonal arrays of particles (OAPs) (Verkman et al., 2011). Disruptions to AQP4 localization have been reported to greatly impair solute transport (Murlidharan et al., 2016; Mestre et al., 2020; Salman et al., 2022), and is a common outcome in a variety of disorders, as well as aging (Verkman et al., 2011; Alvestad et al., 2013; Murlidharan et al., 2016; Palazzo et al., 2019; Kolenicova et al., 2020). AQP4 mislocalization can occur through ablation of α-syntrophin, which facilitates membrane insertion (Mestre et al., 2018), or by interfering with isoform composition, which impedes OAP formation (Palazzo et al., 2019; de Bellis et al., 2021). Understanding how AQP4 organization and anchoring affects its function, as well as how AQP4-interacting proteins are compromised in specific disease states, may help further characterize the routes fluid follows into and out of the brain. Additionally, despite what is known about the significance of periarterial AQP4, studies to date have not examined relative AQP4 expression at these sites compared to venules, which serve as drainage pathways for accumulated water and potential contaminants. Localization of AQP4 on perivenous endfeet and its potential role in fluid drainage mechanisms remain an understudied facet of glymphatic clearance, but could provide key insights about solute clearance routes to limit fluid stagnation within the parenchyma.

### 4.3 Glymphatic system dysfunction in sleep disturbances and “fluidopathies”

Characterization of ISF/CSF distribution routes through the parenchyma, and through individual cells, is a key step in understanding diseases that arise from fluid contamination and disruption to glymphatic clearance. This suite of conditions has been collectively referred to as “fluidopathies” and includes physical injury, hydrocephalus, BBB disruption, stroke, inflammation, neurodegeneration, and glioblastoma (Taoka and Naganawa, 2021; Xu et al., 2021). Because ECS composition and solute transport are significantly affected by behavioral state (Xie et al., 2013; Ding et al., 2016), sleep loss and circadian rhythm disruption may also be considered fluidopathies, even in the absence of other pathological states (Taoka and Naganawa, 2021). Sleep disturbances are often reported in patients with TBI (Tapp et al., 2020), ischemic stroke (Duss et al., 2023), Alzheimer’s disease (AD) (Ju et al., 2017), and many other disorders (Taillard et al., 2021), suggesting that disruptions to sleep might be strongly interlinked with glymphatic dysfunction. The development of a novel method known as “diffusion tensor imaging along the perivascular space” (DTI-ALPS), has enabled non-invasive characterization of glymphatic dysfunction in human patients based on impairments to water diffusion along perivascular spaces (Taoka et al., 2017). DTI-ALPS analysis has revealed significant glymphatic system impairments in patients with normal pressure hydrocephalus (Bae et al., 2021), glioma (Toh and Siow, 2021a), ischemic stroke (Toh and Siow, 2021b), Parkinson’s disease (PD) (Si et al., 2022), and temporal lobe epilepsy (Lee et al., 2022). While sleep can be affected as a secondary consequence of stress, pain, or other symptoms associated with severe illnesses (Nijs et al., 2017), it is also important to consider disordered sleep as a direct consequence of glymphatic dysfunction, as shown by reduced DTI-ALPS indices in REM sleep behavior disorder (Si et al., 2022) and in patients reporting reduced sleep quality (Saito et al., 2023). While these imaging studies provide valuable insights into how CSF is dysregulated in various fluidopathies, it is still unknown whether glymphatic dysfunction is an underlying cause for these disorders, or whether it is merely a symptom.

As astrocytes play a key role in regulating CSF composition and facilitating its transport (Simard and Nedergaard, 2004; Ding et al., 2016), it is important to consider the ways in which reactive glia might contribute to glymphatic dysfunction. Alterations in astrocyte function and morphology in response to a pathological event is known as astrocyte reactivity or reactive gliosis (Pekny and Pekna, 2014; Burda et al., 2016; Escartin et al., 2019, 2021). While the reactive astrocyte population is extremely heterogeneous and there is no single “reactive astrocyte” phenotype, these changes are generally thought to entail alterations in astrocytes’ homeostatic and neurotrophic functions (Escartin et al., 2019; Sofroniew, 2020). Some common patterns observed in astrocytes across disease states include hypertrophy, or increased branching of processes, altered protein expression (including AQP4), disruptions to ionic and neurotransmitter homeostasis (especially K^+^), and increased secretion of immunomodulators, all of which have the potential to affect BBB integrity and, by extension, perivascular space dynamics (Vizuete et al., 1999; Wang and Parpura, 2016; Escartin et al., 2021). Overall, maladaptive astrocyte functions, especially those functions associated with ECS composition and fluid transport, are observed in many of the fluidopathies listed above (Taoka and Naganawa, 2021; Reed and Blazer-Yost, 2022).

Aquaporin-4 plays a key role in facilitating passage of ISF from periarterial spaces into the brain parenchyma (Haj-Yasein et al., 2011; Iliff et al., 2012), and multiple lines of evidence point to AQP4 dysregulation as a key mechanism for fluidopathy formation. Early characterization of AQP4 expression has shown that, in pharmacologically induced models of neurodegeneration, astrocytes that express increased amounts of glial fibrillary acidic protein (GFAP), namely, reactive astrocytes, also express increased levels of AQP4 mRNA (Vizuete et al., 1999). Upregulation of AQP4, which is observed during inflammation, cerebral, and cellular edema, has been shown to exacerbate fluid accumulation both within cells, and in the brain overall, preventing proper drainage of water and potentially toxic metabolites (Aoki et al., 2003; Warth et al., 2007; Alexander et al., 2008; Cao et al., 2012). Similarly, post-mortem human tissue from patients with AD show that increased AQP4 levels and increased distribution away from the endfeet were strongly associated with higher aβ accumulation (Simon et al., 2018). While these findings suggest that interfering with AQP4 might be an attractive target for the resolution of some types of edemas by limiting further water entry into the parenchyma, multiple reports have revealed negative effects of AQP4 KO on glymphatic system function and astrocyte volume dynamics. One group showed that AQP4 KO greatly impeded ISF flow in deep brain structures, like the thalamus and caudate nucleus (Teng et al., 2018). AQP4 knockout also suppresses ISF and CSF exchange across the brain under conditions of hypoosmotic stress (Haj-Yasein et al., 2011), subarachnoid hemorrhage (Liu et al., 2020), and oxygen-glucose deprivation (Chmelova et al., 2019). Whether this is due to a lack of parenchymal influx or a lack of perivenous efflux/drainage is still unknown.

Sleep disruptions have further consequences for glymphatic clearance. Zhang et al. (2020) found that in already sleep-deprived mice, knockout of AQP4 further inhibited CSF flux, reduced metabolite clearance, induced neuroinflammation, and caused memory deficits in adult mice. In addition to reduced AQP4 expression in sleep-deprived mice, the AQP4 that was present was not polarized to the vasculature, rather, it was evenly distributed throughout the sampled tissue (Zhang et al., 2020). In a mouse model of TBI, sleep disruption following injury increased stress, disrupted AQP4 localization, and resulted in a longer lasting inflammatory response (Tapp et al., 2020). In humans, patients with chronic insomnia reported poorer sleep quality and had lower serum levels of AQP4, suggesting impaired glymphatic clearance (Yang et al., 2022). Understanding how water enters cells, and the routes it takes as it passes through and then exits the brain, is a critical step in determining how to relieve edema without trapping excess fluid and toxic metabolites in the ECS and within cells.

While sleep loss alone has negative consequences for AQP4 characteristics and overall brain health (Kitchen et al., 2020), sleep loss combined with AQP4 disruption results in even poorer outcomes for cognitive performance and glymphatic transport (Zhang et al., 2020). Not only would there be reduced entry of fluid from perivascular spaces, but loss of AQP4 would also deprive the astrocyte of an outlet for water, and any water entering the astrocyte through other means would be trapped within the cell. Recent reports suggest that, in a mouse model of TBI, poor sleep and AQP4 dysregulation interact to worsen behavioral outcomes and hinder recovery (Tapp et al., 2020). Alternatively, the upregulation and loss of endfeet polarization of AQP4 could mean that, instead of “used” or “contaminated” ISF being drained out through perivenous spaces, water is instead drained back into the parenchyma, disrupting ion and neurotransmitter homeostasis in the ECS. Both of these would result in cellular and/or cerebral edema, and buildup of toxic metabolites in the ECS. In conditions like ischemic stroke, where AQP4 is overexpressed in astrocyte endfeet (Aoki et al., 2003; Murata et al., 2020), the increased opportunity for water entry could be an underlying factor for astrocytic edema. This could result in failure of astrocytes to shrink to accommodate ECS expansion, which would also hinder glymphatic clearance that typically occurs during sleep. This has been considered as a potential explanation for the profound protective effects of AQP4 block or knockout in such conditions (Manley et al., 2000; Pirici et al., 2017; Chmelova et al., 2019). However, given the detrimental effects of AQP4 for waste accumulation (Iliff et al., 2012; Mestre et al., 2018; Rainey-Smith et al., 2018), caution should be used when investigating AQP4 block or ablation as a clinical strategy. Overall, these data suggest a narrow range for optimal AQP4 facilitation of glymphatic clearance, and disruption of this balance in either direction could have deleterious effects.

While glymphatic system impairment has been reported in numerous disease contexts, only a limited number of studies have examined the direct role of astrocytes in glymphatic clearance routes (aside from AQP4, whose mechanisms have remained controversial). Astrocytes, like other cells, respond to physical changes in the extracellular matrix (ECM) by converting structural changes of the parenchyma into intracellular signals through a process known as mechanotransduction (Chen and Qiu, 2022; Donnaloja et al., 2023). Extreme mechanical stress may be exerted on parenchymal fluid routes in a variety of pathologies, like TBI (Gomez-Cruz et al., 2024), and could potentially contribute to the reduced glymphatic transport observed in fluidopathies. Astrocyte endfeet are particularly sensitive to shear forces of ISF flux driven by vascular pulsatility, especially where the BBB is compromised and flow velocity is reduced (Momin et al., 2021). However, the impact of mechanotransduction and ECM rigidity on astrocyte water transport remains a relatively understudied facet of the glymphatic system. Candidates for glymphatic clearance-related channels beyond AQP4 which may also be altered in disease states include mechanosensitive transient receptor potential (TRP) channels TRPA1 (Trotier et al., 2023) and TRPV4 (Liao et al., 2023), Piezo-type mechanosensitive ion channel component 1 (Piezo1) (Ballesteros-Gomez et al., 2023; Trotier et al., 2023) or even mechanosensitive NMDA receptors (Maneshi et al., 2017). Even fewer studies have investigated solute transport capabilities of reactive astrocytes in the context of sleep-associated fluidopathies. Thus, it remains unknown whether astrocyte fluid dysregulation directly interferes with glymphatic clearance by contributing to CSF/ISF contamination and hindering flow, or whether astrocytic damage and reactivity creates an environment that is more conducive to the development of fluidopathies.

### 4.4 Effects of aging and neurodegenerative disease on glymphatic clearance

The brain undergoes many structural changes over the course of aging that, even without age-related disease or neurodegeneration, can result in deteriorating neuronal health and cognitive decline. Some of these behavioral outcomes, like worsening memory, are thought to be associated with poorer sleep quality reported by the aging population (Voumvourakis et al., 2023). As efficient fluid circulation and waste transport are intimately tied to sleep quality, the impact of aging on glymphatic system function is becoming an increasingly important facet of geriatric research (Iliff et al., 2012; Xie et al., 2013; Kress et al., 2014). The structure of vasculature and perivascular spaces that, in part, drive glymphatic clearance may also be affected by disease, as well as aging (Iliff et al., 2013; Jessen et al., 2015). Fico et al. (2022) reported that pulsatility of cerebral vasculature is reduced in otherwise healthy aged adults, suggesting a decreased driving force for fluid throughout the brain. Aging brains are characterized by elevated and persistent levels of inflammation (Jiang and Cadenas, 2014; Gordleeva et al., 2020), meaning that astrocytes are also more likely to express reactive phenotypes, such as hypertrophy, GFAP upregulation, and AQP4 dysregulation (Cotrina and Nedergaard, 2002; Zeppenfeld et al., 2017; Clarke et al., 2018; Palmer and Ousman, 2018). Given these impacted functions, we may hypothesize that, much like other fluidopathies, age-related deterioration likely affects astrocytic ion and water homeostasis in a way that impacts their capacity for glymphatic clearance (Syková et al., 2000).

Aging dramatically increases risk for neurodegenerative disorders, many of which feature some degree of fluidopathy, such as AD, PD, and dementia (Kress et al., 2014; Wang and Mourrain, 2020; Taillard et al., 2021). A common feature of neurodegenerative disease is the aggregation of misfolded proteins, and aβ has been used as the primary biomarker for CSF clearance and glymphatic efficacy (Iliff et al., 2012; Bondareff, 2013; Kress et al., 2014; Ju et al., 2017; Mestre et al., 2018; Simon et al., 2018; Zhang et al., 2020; Silva et al., 2021). Taken together, the findings from these studies show that increased aβ accumulation is often a result of poor CSF/ISF transport, which is associated with worsened sleep, as well as dysregulated AQP4 expression and localization. Buildup of toxic metabolites also includes components associated with aβ plaques, including amyloid precursor protein (APP) and Tau (Zhang et al., 2020; Silva et al., 2021). Insufficient sleep further drives accumulation of aβ by reducing opportunities for clearance of toxic metabolites (Gordleeva et al., 2020). This finding has even been replicated in humans, with Ju et al. (2017) reporting that specific disruption of SWA, even for one night, is strongly associated with increased aβ.

In summary, sleep homeostasis, glymphatic clearance, and healthy astrocytes are all vital for maintaining normal brain function. Conversely, disordered sleep, CSF contamination and stagnation, and astrocyte malfunction have the potential to interact to cause significant decline in overall brain health. Many of these homeostatic functions are compromised both during healthy aging and in neurodegenerative disease. It is yet unknown whether sleep deprivation or disruption creates an environment that is conducive to astrocyte reactivity and subsequent cognitive decline, or whether the reprogramming of astrocytes to a disordered state causes alterations in neuronal activity leading to sleep disruptions. It could be a combination of both, wherein one feeds into the other to amplify development of pathology.

## 5 Astrocytic control of immunomodulators in physiological and pathological conditions

In addition to the ions and neuromodulators that circulate throughout the brain’s interstitial space, CSF also carries various immunoactive substances that serve important roles in the sleep-wake cycle (Besedovsky et al., 2019). It has been commonly reported, both anecdotally and in regulated experimental environments, that infection and disease are often accompanied with abnormal sleep experiences (Pollmächer et al., 2002; Besedovsky et al., 2019). The converse is also true: in both human and animal models, sleep deprivation and disruption, even in the short term, has been reported to induce neuroinflammation (Nijs et al., 2017; Manchanda et al., 2018; Garofalo et al., 2020). Studies in both human and animal models have identified multiple cytokines whose concentrations fluctuate in a circadian pattern, and whose circulation is critical to the initiation and maintenance of sleep (Pappenheimer et al., 1975; Krueger et al., 1990; Pollmächer et al., 2002; Wilson et al., 2002). Astrocytes endogenously express receptors for somnogenic cytokines, which when blocked, have functional consequences for sleep (Opp et al., 1992; Figure 2). Astrocytes have also been identified as significant sources of IL (interleukin)-6 (Frei et al., 1989; Norris and Benveniste, 1993), TNFα (tumor necrosis factor α), and IL-1 (Yu and Lau, 2000; Vezzani et al., 2008). This suggests that astrocytes can contribute to immune signaling even in the absence of injury, pathogen, or other immune triggers (Wilson et al., 2002). Numerous studies have observed that neuroinflammation resulting from sleep disruption causes direct activation of astrocytes, as well as indirect astrogliosis through the activation of microglia (Hsu et al., 2003; Bellesi et al., 2017; Liddelow et al., 2017; Manchanda et al., 2018; Drew et al., 2023). Conversely, treatment with LPS, a robust instigator of systemic inflammation, has been shown to cause dramatic shifts in sleep architecture by increasing the ratio of NREM to REM sleep, a sign of sleep disruption (Krueger et al., 1986; Moldofsky et al., 1986; Lancel et al., 1995). Using this framework, it can be considered that immune responses and sleep disruptions *together* form a positive feedback loop, in which each condition aggravates the other. Given the role of astrocytes in the brain immune response and in the facilitation of sleep, these cells may hold clinical significance as an exit pathway from positive feedback of sleep disruption.

### 5.1 Somnogenic cytokines and their effects on sleep induction and duration

The observation that increased amounts of slow-wave sleep accompanying the febrile response to bacterial pyrogens led researchers to focus their efforts on substances that trigger immune responses, namely the class of peptides known as cytokines (Krueger et al., 1990). Interleukins (IL) are a subset of cytokines that have specifically been shown to trigger an immune response both directly and indirectly, by initiating other signaling cascades (Krueger et al., 1990, 1998). Early reports have shown that administration of human endogenous pyrogen (an early name for IL-1) into rabbit cerebral ventricles caused prolonged periods of SWS under a mechanism that occurs concurrently with a fever response (Krueger et al., 1984). These findings were replicated by a study administering astrocyte-derived IL-1, which showed that SWS was enhanced at the expense of REM sleep (Tobler et al., 1984). An important extension of this finding is that block of the fever response still resulted in extended SWS, emphasizing that the role of cytokines is independent from body temperature effects on sleep architecture (Krueger et al., 1984). To extend these correlational findings, a subsequent study found that specific activation of the IL-1β receptor in rats and rabbits potentiated SWS, and that antagonism of the IL-1β receptor blocked or reduced these effects (Opp et al., 1992). These sleep-promoting effects appear to be attenuated by anti-inflammatory cytokines, such as interleukins-4, 10, and 13, which actively repress activity of pro-inflammatory cytokines and inhibit NREM sleep (Kushikata et al., 1999; Kubota et al., 2000; Besedovsky et al., 2019). For example, administration of IL-13 and TGF (transforming growth factor) into rabbit cerebral ventricles inhibited NREM sleep (Kubota et al., 2000). While a mechanistic explanation for this has yet to be explored, the authors suggested that anti-inflammatory cytokines may promote sleep either through the direct inhibition of sleep-promoting factors, or through the production of sleep inhibitory substances (Kubota et al., 2000).

These findings suggest that the actions of certain cytokines are consequential for sleep, especially slow-wave activity that accompanies NREM sleep. The concentrations of these cytokines may build up as a result of increased wakefulness, as do the somnogens discussed above, but evidence also suggests circadian fluctuations of sleep-promoting cytokines (Norris and Benveniste, 1993; Krueger et al., 1998; Duhart et al., 2013). IL-1 levels in the plasma peak immediately preceding SWS onset (Moldofsky et al., 1986). Subsequent experiments showed that, in addition to IL-1 (specifically IL-1β), increases in TNFα and IL-6 activity were also strongly correlated with SWS (Krueger et al., 1998; Vgontzas et al., 1999). Despite these findings, increased understanding of the sleep-promoting effects of cytokines in physiological concentrations remains an ongoing area in need of further study.

As major participants in CNS immune signaling, astrocytes secrete cytokines in response to various immuno- and neuromodulators, some of which exert robust sleep-promoting effects (Wilson et al., 2002; Sofroniew, 2014; Figure 2). High concentrations of NE, which has been shown to build up as a consequence of sleep deficiency, can directly cause astrogliosis (Griffith and Sutin, 1996), cause increased protraction of astrocyte processes into the ECS (Sherpa et al., 2016), and, in extreme cases, trigger both small and pathological elevations in IL-6 (Norris and Benveniste, 1993). Immune factors are also able to directly influence circadian activity within astrocytes, with Duhart et al. (2013) showing that TNFα activation of astrocytes in the SCN alters expression of proteins that regulate their own internal clocks, which could have significant consequences for phase shifts within sleep, as well as overall state changes between sleep and wakefulness. On the other hand, melatonin, a robust sleep-promoting hormone, has a neuroprotective effect in attenuating astrogliosis (Babaee et al., 2015; Yawoot et al., 2022; Dorranipour et al., 2024) and associated immune responses in animal models of TBI, obesity, and hypoxia (Kaur et al., 2008; Babaee et al., 2015; Dorranipour et al., 2024). Importantly, it has also been shown to reduce edema (Kondoh et al., 2002; Li et al., 2014), potentially through interfering with AQP4-mediated swelling (Li et al., 2014). Other somnogenic substances, like nitric oxide and histamine, have also been examined for their contributions to the immune response and, independently, their role in the sleep-wake cycle (Chao et al., 1996; Krueger et al., 1998; Hsu et al., 2003). However, the specific participation of astrocytic immunomodulatory signaling in the context of sleep has yet to be thoroughly explored.

Another class of cytokines influencing sleep, prostaglandins (PG), are thought to act as secondary inflammatory mediators in response to other cytokines (Krueger et al., 1990; Pentreath et al., 1990). These molecules enable communication between systemic immune responses and cerebral endothelial cells, especially during episodes of fever (Kis et al., 2006). One subtype of prostaglandins, PGD_2_, is widely considered to have sleep-promoting effects (Ueno et al., 1983; Krueger et al., 1990). Continuous administration of PGD_2_, but not PGE_2_, in rats has been shown to increase the amount of time spent in NREM sleep (Ueno et al., 1983). Another group replicated these results with the added finding that, despite altered NREM:REM ratio, the NREM sleep was physiologically indistinguishable from naturally occurring sleep (Hayaishi et al., 2004). Recent work investigating the mechanisms underlying the sleep-promoting effects of PGD_2_ has shown that PGD_2_-mediated adenosine release activates sleep-promoting neurons within the VLPO (Scharbarg et al., 2023). Disruption of PG signaling can have significant impacts on sleep architecture. Mice lacking the ability to produce PGs fail to show homeostatically increased periods of NREM sleep following sleep deprivation (Matsumura et al., 1991; Hayaishi et al., 2004). Astrocytic expression of transporters for PGs have been shown to increase when stimulated by LPS, further facilitating passage of PGs across the BBB and into the brain parenchyma (Kis et al., 2006; Tachikawa et al., 2012). Under the combined effects of ATP and the cytokines IL-1β, TNFα, and IFNγ (interferon γ) (which each have their own well-documented somnogenic effects), the precursors for PG production in astrocytes were markedly increased (Xu et al., 2003). A summary of somnogens in the brain extracellular space is provided in Table 1.

**TABLE 1:** Summary of somnogenic factors in the ECS and their effects on sleep and astrocyte function.

Somnogenic factors	Role in behavioral state	Pathological consequences when dysregulated	Main sources
Norepinephrine	– Promotes wakefulness and arousal – Increases extracellular K^+^ – Modulates astrocyte and ECS volume	– Astrogliosis – Increased cytokine production	Neurons (Locus Coeruleus)
Adenosine	– Promotes sleep – Inhibits wake–promoting circuitry – Elevated prior to sleep	– Astrogliosis	Astrocytes, neurons (ATP metabolite)
Melatonin	– Promotes sleep	– Alleviates astrogliosis, edema caused by spinal cord injury, ischemia, **others?**	Pineal Body
Histamine	– Promotes wakefulness	– Increased cytokine production	Neurons (hypothalamus)
Dopamine	– Promotes arousal	– Loss of dopaminergic neurons contributes to PD (inflammation and reduced glymphatic efficacy)	Neurons (hypothalamus, brainstem)
Orexin	– Promotes arousal	– Loss of orexinergic neurons contributes to PD (inflammation and reduced glymphatic efficacy)	Neurons (hypothalamus)
Calcium (Ca^2+^)	– Elevated during sleep	– Increased BBB permeability	Neurons, choroid plexus/vasculature
Potassium (K^+^)	– Elevated during wake – Decreased during sleep – Elevations in K^+^ cause astrocyte swelling	– Hyperexcitability and excitotoxicity	Astrocytes, neurons, choroid plexus/vasculature
IL–1	– Promotes NREM sleep	– Astrogliosis – Promotes inflammation – Upregulates AQP4 – Exacerbates edema caused by ALF, **others?**	Astrocytes, microglia
IL–6	– Promotes sleep	– Astrogliosis – Promotes inflammation – Exacerbates edema caused by ALF, **others?**	Astrocytes, microglia
IL–13	– Inhibits sleep	– Anti–inflammatory	Microglia
TNFα	– Promotes NREM sleep – Upregulated in sleep deprivation	– Astrogliosis – Promotes inflammation – Exacerbates edema caused by ALF, **others?**	Astrocytes, microglia
PGD_2_	– Promotes NREM and REM sleep – Increases adenosine levels	– Astrogliosis – Increased BBB permeability	Mast cells (choroid plexus)
PGE_2_	– Inhibits sleep	– Astrogliosis	Astrocytes, microglia
NO	– Promotes sleep – Triggers adenosine release	– Astrogliosis – Neuronal damage	Neurons (NOS)

While the concentrations of these factors fluctuate over the course of the sleep-wake cycle, pathological overproduction, upregulation, or depletion of these can have detrimental consequences for astrocytic function. Overall, there are numerous substances whose direct contributions to astrocyte volume changes remain unknown. It is important to note that this is not an exhaustive list of all somnogenic factors that may be involved in the astrocytic modulation of sleep. IL, interleukin; TNF, tumor necrosis factor; PGD, prostaglandin; NO, nitric oxide; NOS, nitric oxide synthase.

Astrocytes are directly involved in the signaling pathways associated with the cytokines affecting sleep discussed above (see Sofroniew, 2014 for a thorough review). The same substances that may act as somnogens in a homeostatic context may, in a pathological state, alter astrocyte function in a manner that dramatically reshapes sleep patterns both acutely, and over the long term. These functions include regulation of ECS volume, turnover of neuromodulators and somnogenic cytokines, and transport of fluid. Are these merely parallel phenomena that occur coincidentally, or can one influence the other? Do circulating immunomodulators, both those that fluctuate on a circadian basis, and those that build up during standard periods of wakefulness, contribute to the acute astrocytic structural changes that are observed across states? An immune response triggered by LPS alone has been shown to be sufficient to cause cerebral edema (Alexander et al., 2008; Cao et al., 2012). Astrocytes possess the receptors and intracellular signaling molecules necessary for responding to cytokines (Wilson et al., 2002; Vezzani et al., 2008; Sofroniew, 2014), but it is unknown whether they are only able to respond to these substances if they are highly concentrated, or if they can also undergo structural changes to constitutively circulating levels that occur physiologically. Opp et al. (1992), for example, found that a 12 nanomole dose, but not a 6 nanomole dose of human IL-1β administered to rabbits produced a robust increase in the amount of time spent in NREM sleep. However, it is difficult to ascertain whether this administration represents a pathological increase, and what this increase might mean for astrocyte function. Additionally, it is difficult to determine what *would* constitute a pathological increase, given species differences, the diversity of ECS composition across brain regions, and the currently available methodologies for sampling ECS *in vivo*. Observations from various pathogenic models, especially those in cases of cellular or cerebral edema, may provide some insight into how neuroinflammation and maladaptive astrocyte swelling or shrinking may interact to alter sleep architecture.

### 5.2 Effects of sleep disruption on cerebral immune function

Pharmacological or pathological disruptions to cytokine signaling have been repeatedly shown to interfere with normal sleep, providing important clues as to how an overactive immune response may increase risk of developing sleep disturbances or sleep loss (Besedovsky et al., 2019; Irwin, 2019). Astrocytes already affected by sleep disruption may create an environment that facilitates development of other pathologies, which can in turn have adverse consequences on the homeostatic functions of astrocytes. Astrocytes that undergo this “reprogramming” are said to become reactive, and display a wide range of functional and morphological alterations that distinguish them from their healthy counterparts (Pekny and Nilsson, 2005; Escartin et al., 2021). This state is extremely diverse with respect to disease type, severity, and progression, but can manifest as changes in GFAP expression, uncoupling/loss of communication via gap junctions, recruitment of proinflammatory cytokines, an increase in BBB permeability, loss of ion buffering capabilities, proliferation and formation of glial scars (most typically in cases of tissue damage), aberrant calcium signaling, and hypertrophy (Pekny and Nilsson, 2005; Kuchibhotla et al., 2009; Sofroniew, 2020). Decrease of extracellular Ca^2+^ or depletion of intracellular Ca^2+^ stores may cause disruption to the BBB, making it more permeable to infiltrating cytokines and disrupting the neurovascular unit (Shigetomi et al., 2019). Uncoupling of reactive astrocytes results from the loss of connexins that span the gap junction that joins two astrocyte processes together (Williamson et al., 2021). All these outcomes can lead to reduced buffering and redistribution of small molecules and ions, such as K^+^, suggesting that reactive astrocytes impact the ECS composition differently than healthy ones.

There is ample evidence that sleep deprivation is detrimental to brain function and overall health, creating a pro-inflammatory environment facilitating further sleep loss (Zhu et al., 2016; Nijs et al., 2017; Wadhwa et al., 2017). Initial experiments characterizing the effects of sleep deprivation on immunomodulators in rodent serum revealed a significant increase in the sleep-promoting and pro-inflammatory cytokines IL-1, TNFα, and IL-6 (Hu et al., 2003). An important consideration in these experiments is that the observed effects were a result of sleep deprivation specifically, as non-sleep related physical stress did not produce these same effects (Hu et al., 2003). These findings are extended in models of chronic sleep deprivation (less than 6 h of sleep over a 24-h period), which show increased levels of inflammatory cytokines (Manchanda et al., 2018), worsened performance on cognitive measures (Manchanda et al., 2018; Zhang et al., 2020), and enhanced activation of astrocytes and microglia (Kim et al., 2014; Bellesi et al., 2017; Manchanda et al., 2018; Drew et al., 2023). Even sleep fragmentation, which does not detract from overall sleep duration or alter sleep architecture, activates an immune response that alters cognitive abilities and negatively impacts subsequent sleep (Ramesh et al., 2012). Taken together, these findings suggest that while disruption of cytokine signaling may have profound effects on sleep onset and sleep duration, sleep loss itself has the potential to reciprocally dysregulate astrocytic cytokine secretion.

The astrocyte marker GFAP has been shown to be instrumental in defining astrocyte structure and alterations in its expression have been used as a classic hallmark of reactive astrocytes. Most often observed is an increase in GFAP expression which may be a sign of hypertrophy or increased branching of astrocyte processes (Escartin et al., 2021). In the healthy rodent SCN, GFAP expression has been shown to fluctuate rhythmically over 24 h periods, even when deprived of external cues (Lavialle and Servière, 1993). Interestingly, astrocytes also display an aberrant phenotype in response to sleep disruption exhibited by GFAP upregulation (Hsu et al., 2003). Proteomic analysis of astrocytes in sleep-promoting areas, like the VLPO of the hypothalamus, shows that sleep deprivation can increase the number of reactive astrocytes (Kim et al., 2014), suggesting that sleep loss can lead to proliferative astrogliosis, considered to be an extreme reactive astrocyte response typically observed in the formation of glial scars (Sofroniew, 2014; Zhang et al., 2020). Increased branching or enlargement of reactive astrocyte processes could reasonably be expected to reduce the volume of the ECS, which could prove consequential for sleep-wake homeostasis by increasing concentrations of circulating cytokines, ions, and neurotransmitters, and hindering their movement. Sleep loss has been shown to result in increased interactions between astrocytic processes and synapses (Bellesi et al., 2015), placing astrocytes in a position to either facilitate return to homeostasis or contribute to detrimental loss of supportive functions. The characterization of astrocyte structural changes in conditions of sleep deprivation have yet to be fully explored and so the consequences of these alterations remain speculative.

### 5.3 Edema, inflammation, and sleep disruption

As discussed above, multiple disease states may feature, or even arise from, dysregulation and contamination of fluid within the brain (Taoka and Naganawa, 2021). Cellular edema (also known as cytotoxic edema) is typically associated with pathological conditions that result from ionic imbalances and associated fluid movement into cells (Stokum et al., 2015). Cerebral edema is a multifaceted form of fluid accumulation that is said to arise from the combined effects of vasogenic edema, the mass exodus of ions and other solutes from perivascular spaces, and cytotoxic edema (Stokum et al., 2016; Sepehrinezhad et al., 2020). All edema subtypes result from a wide array of pathological triggers that create unique profiles of swelling and neuroinflammation which can also induce varying forms of reactive gliosis (Sofroniew, 2014; Escartin et al., 2021). These changes are also distinct from hypertrophy and remodeling of processes, which often occurs in reactive astrogliosis, a state that likely often accompanies cerebral edema due to tissue damage and/or inflammation.

Cellular edema mainly results from ISF being driven into cells from the neuropil, while CSF is responsible for the overall tissue swelling characteristic of cerebral edema (Iliff and Simon, 2019; Mestre et al., 2020). However, much of the literature surrounding these conditions does not clearly distinguish between the two, and it is unclear how they might differ from one another in a pathological context. For example, the breakdown of the physical barriers that normally separate ISF from CSF may cause CSF stagnation, or otherwise compromise the functionality of the glymphatic system (Benveniste et al., 2017). Due to this ambiguity, throughout this section we will use “fluid” as a general term to refer to the water and solutes occupying the ECS. Edematous astrocytes are key effectors of fluid composition within the diseased or damaged brain, and thus represent a key target in understanding why sleep disorders in various brain pathologies have such high comorbidities. Much of the evidence for the detrimental effects of astrocytic edema on sleep comes from studies on ischemic stroke, which results from blocked perfusion of blood and oxygen to the brain (Mestre et al., 2020). Cytotoxic edema and astrogliosis are well-documented hallmarks of the brain damage that results from an ischemic event (Pivonkova and Anderova, 2017; Menyhárt et al., 2022; Tang et al., 2022). Reactive astrocytes are known to produce numerous cytokines, many of which participate in somnogenic signaling (Krueger et al., 1990; Sofroniew, 2014). Sleep disturbances have been observed in both animal models of ischemic stroke, and in human patients (Sharma et al., 2022; Duss et al., 2023), although the mechanisms underlying them are not well established.

Given the importance of fluid composition in sleep-wake transitions, we can speculate that the altered expression of channels and transporters on edematous reactive astrocytes could be one potential source of ionic imbalance that leads to sleep disruption. Potassium regulation is an extremely critical function of astrocytes (Kofuji and Newman, 2004), and the loss of GJ coupling combined with failure of the NKA could cause K^+^ levels to rise far above baseline levels, which aside from being neurotoxic could prevent K^+^ from falling to the levels required to facilitate a state shift into sleep (Ding et al., 2016; Forsberg et al., 2022). Elevated K^+^ levels themselves have been consistently shown to cause astrocytic swelling as K^+^ and water accumulate in the cell (Pasantes-Morales and Schousboe, 1989; Walch et al., 2020), generating a feedback loop of cellular edema and excitotoxicity that could exacerbate disruptions in sleep-wake homeostasis. Additionally, failure of excess K^+^ to clear away could also contribute to a persistently enlarged ECS, which is characterized by significantly reduced glymphatic clearance (Xie et al., 2013). The daily fluctuations in K^+^ and adrenergic receptor activation are associated with the routine, readily reversible astrocytic volume changes that facilitate glymphatic clearance during sleep (Xie et al., 2013; Sherpa et al., 2016). Extending these findings to pathological conditions, we may hypothesize that prolonged adrenergic receptor activation and resultant K^+^ elevations may accompany extended cellular edema, reduced solute transport, and poorer sleep outcomes. Accordingly, inhibition of adrenergic signaling has been shown to resolve edema and improve glymphatic clearance in a mouse model of TBI (Hussain et al., 2023), as well as restore K^+^ homeostasis in ischemic stroke (Monai et al., 2019, 2021). While measurements of potential sleep disruptions were beyond the scope of these studies, they are a frequently observed symptom of fluidopathies (Taoka and Naganawa, 2021), and so warrant further investigation. Additionally, these studies utilized general antagonism of adrenergic receptors, preventing assessment of astrocytic adrenergic signaling specifically. This is noteworthy considering that (reactive) astrocytes in these conditions are significant mediators of altered K^+^ homeostasis and tissue edema (Stokum et al., 2016; Monai et al., 2021).

The involvement of astrocytes in cellular and cerebral edema suggests a bidirectional interaction – excess fluid in the ECS has the potential to be osmotically driven into individual cells, and astrocytes possess mechanisms that allow them to expel ions and neuroactive substances back into the ECS through volume regulatory processes. What happens, then, when astrocyte volume regulatory functions are compromised as a result of the inflammatory environment associated with edema? Given the frequent comorbidities of both types of edema and sleep disruptions (Besedovsky et al., 2019; Garofalo et al., 2020; Duss et al., 2023), the combined effect of edema and neuroinflammation could have significant negative consequences on astrocyte contributions to sleep architecture by altering their ability to regulate ECS volume and composition. However, there remain relatively few studies that specifically investigate maladaptive astrocyte volume regulation as a *direct* cause of, or *direct* result from, sleep deprivation and associated neuroinflammation.

The interface between astrocytic edema and neuroinflammation is also explored in studies of hepatic encephalopathy (HE), which features astrogliosis and cell swelling that is exacerbated by a peripheral immune response and oxidative stress (Jayakumar et al., 2014; Sepehrinezhad et al., 2020). HE may occur as a symptom of acute liver failure (ALF), or from more severe stages of cirrhosis, and commonly features sleep disturbances (Wright et al., 2007; Elsherbini et al., 2022). In this condition, the predominant threat to brain health arises from failure of the liver to filter out ammonia from the plasma, which results in its infiltration into the brain parenchyma and the formation of an ionic gradient that drives fluid into astrocytes (Sepehrinezhad et al., 2020). Interstitial concentration of ammonia (and by extension, pH) is tightly regulated by astrocytes and plays an important role in the BBB (Sepehrinezhad et al., 2020).

Overall, astrocyte volume regulation is critical in regulating CSF volume and composition across the sleep-wake cycle (Xie et al., 2013; Ding et al., 2016; Sherpa et al., 2016; Chmelova et al., 2019), a balance which is often disrupted in pathogenic states caused by injury, inflammation, and sleep deprivation. Astrocyte fluid dysregulation could serve as a linking mechanism between conditions of cellular edema, neuroinflammation, and associated sleep disturbances. Fluid accumulation in any space of the brain can have drastic consequences for overall brain health by increasing intracranial pressure, disturbing the osmotic balance, and causing herniations within the brain, all of which could lead to significant tissue damage (Wang and Parpura, 2016). As discussed earlier, hypertrophy without fluid accumulation could still affect ECS volume and composition to sufficiently disrupt sleep onset and sleep maintenance. While the relationship between cellular edema and hypertrophy is still unclear, the resulting changes to solute, ion, and immunomodulator concentrations could be highly influential to sleep-wake transitions and sleep architecture.

## 6 Discussion

In summary, astrocytes are critical effectors of ECS volume and composition, including ion homeostasis, neurotransmitter concentration, CSF/ISF transport, and immunomodulators, all of which can impact cognitive function and overall brain health. Fluid contamination and stagnation, sleep disruption, inflammation, and reprogrammed astrocyte function are all implicated in a multitude of diseases and disorders, although it has been difficult to establish causal relationships between these dysfunctional states. Where past focus has been attributed mainly to neuronal activity, astrocytes are now being incorporated into current frameworks of understanding sleep mechanisms and pathologies.

A number of factors should be considered when interpreting sleep research. First, it is important to note that “sleep” and “wake” themselves are not binary categories – there is variation within each state with respect to levels of arousal. The underlying neuronal activity between “awake and at rest” and “awake and active,” for example, could arise from significantly different ECS profiles (Saper et al., 2010). Usually, sleep in experimental conditions is defined by either REM or NREM sleep, which means that comparisons between wake, NREM, and REM sleep, as well as transitional periods between states, remain an understudied aspect of ECS dynamics. Further complicating this matter are the physiological sleep stages compared to the various methods of inducing sleep experimentally. In mouse research, sleep is hard to disentangle from anesthesia-induced unconsciousness, usually due to experimental constraints that prevent the mouse from initiating and maintaining sleep without stress from the experiment. There is still great debate in the literature about the effects of anesthesia, and whether this can be truly comparable to a sleep state. Many real-time *in vivo* measurements of CSF dynamics cannot be conducted in such a way that the animal can maintain natural sleep. In addition, there appears to be a great amount of variability in terms of the methods used to measure sleep. Xie et al. (2013) showed that effects of ketamine/xylazine anesthesia on CSF flow are comparable to sleep, while Hablitz et al. (2019) showed that xylazine, an α2 adrenergic receptor agonist, is associated with increased SWA and more pronounced glymphatic clearance, while isoflurane anesthesia suppresses both delta power and glymphatic clearance.

Last, common measures of ECS volume do not directly examine the volume contribution of astrocytes specifically, nor do they examine the influence of somnogens on astrocyte morphology (Table 1). Rather, they are an indirect quantification of ECS volume. Historically, one of the main challenges with cellular and extracellular volume dynamics has been measurement of volume changes in real time. While much of the evidence points to astrocytes as a key cell type underlying state-dependent ECS fluctuations, methodological constraints prevent establishing a direct causal relationship between the two. A method of probing the volume changes of individual astrocytes *in vivo* and in real time along with state changes would help close this gap. Recently, imaging technology has evolved to allow for higher resolution imaging of ECS, as well as the finer astrocyte processes that infiltrate it, in real time (Tønnesen et al., 2018; Arizono et al., 2021; Arizono and Nägerl, 2022; Denizot et al., 2022). If applied in conjunction with behavioral assays and/or *in vivo* electrophysiology, these techniques could reveal valuable insights into astrocyte volume and ECS dynamics in synchrony with measures of cortical activity. New imaging techniques are also being applied in human patients in order to evaluate the impact of diseases and disorders on sleep quality and other aspects of brain health, like vascular integrity, fluid transport, and cognitive function (Taoka et al., 2017). These advances could help address vital outstanding questions regarding the role of astrocytes in sleep, and in the development of disorders associated with sleep and fluid dysregulation.

## Author contributions

SS: Conceptualization, Writing – original draft, Writing – review & editing. KC: Visualization, Conceptualization, Writing – original draft, Writing – review & editing. WD: Conceptualization, Writing – original draft, Writing – review & editing. TF: Conceptualization, Writing – original draft, Writing – review & editing.
